# Progesterone Resistance in PCOS/PMOS: Molecular Insights and Therapeutic Perspectives

**DOI:** 10.3390/life16101622

**Published:** 2026-09-28

**Authors:** Ekaterina O. Golubenko, Maria V. Poznyak, Sergey A. Timofeev, Evgeniya A. Svidinskaya, Yuri V. Meksha, Vasiliy B. Osadchev, Dmitriy M. Belousov, Timur N. Rashidov, Sergey N. Rusanov, Gelia N. Gildeeva, Ksenia Yu. Meksha, Raisa A. Chilova

**Affiliations:** 1 Department of Obstetrics and Gynecology No. 1, Sechenov University, 119991 Moscow, Russia; 2 Department of Life and Safety and Disaster Medicine, Sechenov University, 119991 Moscow, Russia; 3 Department of Management and Organization of Drug Supply, FPDE, Sechenov University, 119991 Moscow, Russia

**Keywords:** female infertility, reproductive endocrinology, polycystic ovary syndrome, polycystic ovary morphology and metabolic syndrome, progesterone resistance, endometrial receptivity, decidualization, personalized therapy

## Abstract

**Background**: Polycystic ovary syndrome (PCOS), recently renamed as Polycystic Ovary Morphology and Metabolic Syndrome (PMOS), is a leading cause of female infertility. Despite successful ovulation induction treatment, many patients still fail to achieve pregnancy. This discrepancy points to impaired endometrial receptivity, to which progesterone resistance is a contributing factor within a multifactorial pathogenesis. This review explores the molecular, clinical, and therapeutic aspects of progesterone resistance in PCOS/PMOS to improve patient management. **Methods**: A comprehensive literature search of PubMed, Scopus, and Web of Science was conducted, identifying 68 relevant studies for this review. **Results**: The negative effects of progesterone resistance are caused by several interacting factors. Chronic hyperinsulinemia traps the essential progesterone receptor co-activator FOXO1 in the cytoplasm. Additionally, high androgen levels lead to an overexpression of androgen receptors, which compete for shared cellular resources. Furthermore, chronic inflammation and M1 macrophage polarization disrupt the local immune balance, causing decidual cell senescence. These defects are also maintained by epigenetic changes. **Conclusions**: Overall, administering higher doses of progesterone is not sufficient to overcome these underlying barriers. Therefore, this review underlines the need for metabolically targeted treatments, such as lifestyle modifications, metformin, and inositols, which may improve the metabolic and endocrine environment associated with impaired endometrial responsiveness, although direct reversal of progesterone resistance requires further clinical validation.

## 1. Introduction

Infertility is a growing global health issue, affecting approximately 17.5% of the adult population worldwide [1]. Endocrine disorders are among the leading causes of female infertility, with polycystic ovary syndrome (PCOS) being the most common contributor to anovulatory infertility. PCOS affects 8–13% of women of reproductive age and is frequently accompanied by metabolic dysfunction, such as insulin resistance and obesity [2,3]. However, even when ovulation is successfully restored or achieved, pregnancy and live birth rates often remain lower than expected. This clinical paradox points to underlying defects in endometrial receptivity, in which progesterone resistance plays a major role [4].

The understanding of PCOS has changed significantly over the past century. The condition was originally described in 1935 by Stein and Leventhal as a purely gynecological disorder characterized by anovulation and bilateral polycystic ovaries [5], which led to early surgical treatments like ovarian wedge resection [6]. Later, as the roles of gonadotropins and steroidogenesis became clearer, clinical focus shifted toward neuroendocrine regulation, establishing clomiphene citrate as the standard first-line treatment for ovulation induction [7]. During the 1980s and 1990s, the recognition of hyperinsulinemia and metabolic dysfunction led to another paradigm shift, redefining PCOS as a systemic metabolic disorder and introducing insulin-sensitizing agents like metformin and inositols into treatment protocols [8] (Table 1).

Despite these therapeutic advancements, success rates in assisted reproduction for patients with PCOS remain low. Even with the transfer of high-quality embryos, implantation rates fail to match those seen in healthy, fertile controls [4,9]. This highlights the need to look beyond ovarian function and address the systemic nature of the disease. As a result, the international scientific community officially renamed PCOS to Polycystic Ovary Morphology and Metabolic Syndrome (PMOS) [10].

While advances in assisted reproductive technology (ART) have significantly improved outcomes for patients with tubal and male factor infertility, those with PCOS/PMOS often experience a different clinical trajectory. Large-scale clinical data indicate that despite yielding a higher number of retrieved oocytes and forming morphologically top-quality embryos during IVF/ICSI cycles, women with PCOS frequently demonstrate higher rates of early pregnancy loss. A comprehensive meta-analysis of over 10,000 IVF pregnancies demonstrated that patients with PCOS have a 59% higher risk of total miscarriage (RR = 1.59; 95% CI 1.45–1.75) compared to controls, who predominantly represented tubal factor infertility. Importantly, this increased risk was consistent across both preclinical (RR = 1.59) and early clinical (RR = 1.44) miscarriages [11]. This mathematical discrepancy strongly suggests that the primary bottleneck in achieving a live birth for these patients lies not solely in oocyte competence but rather in a hostile or non-receptive endometrial environment.

Under this new definition, progesterone resistance—a condition where the endometrium fails to respond properly to progesterone despite adequate hormone levels—deserves attention as a contributing factor among the multiple mechanisms of endometrial dysfunction [4]. Early microarray studies by Giudice and colleagues demonstrated that women with PCOS exhibit specific molecular disruptions in the mid-secretory phase, regardless of prior ovulation induction [12]. Studies by Li and Feng further highlighted the dysregulation of progesterone receptor isoforms in endometrial stromal cells as a main cause of this resistance [13].

Overall, this review examines the current molecular, clinical, and therapeutic evidence regarding endometrial progesterone resistance in PCOS/PMOS. By highlighting these underlying mechanisms, this review aims to support the development of targeted, personalized strategies to improve reproductive outcomes. This review is limited in scope and addresses endometrial progesterone resistance exclusively in the context of reproduction, without considering other progesterone-related pathologies such as endometrial hyperplasia or endometrial cancer.

## 2. Materials and Methods

For this narrative review, a comprehensive literature search was performed using the PubMed, Scopus, and Web of Science databases. The search included publications without time restrictions and used combinations of Medical Subject Headings (MeSH) and keywords. The search terms included (“polycystic ovary syndrome” OR “PCOS” OR “PMOS” OR “polycystic ovary morphology AND metabolic syndrome”) AND (“progesterone resistance” OR “progesterone receptor” OR “endometrial receptivity” OR “decidualization”) AND (“implantation” OR “fertility” OR “pregnancy outcome”). In addition, the reference lists of relevant reviews and original articles were manually screened to identify studies not captured in the initial database search.

Inclusion criteria were limited to original studies, reviews, meta-analyses, and clinical guidelines published in English. Selected articles had to focus on the molecular mechanisms of progesterone resistance, endometrial receptivity, and therapeutic approaches in PCOS/PMOS.

The source selection involved two stages: an initial screening of titles and abstracts for relevance, followed by a comprehensive review of full texts against the inclusion criteria. It is important to note that this search strategy was intended to support a narrative review of the molecular mechanisms and therapeutic strategies for progesterone resistance, rather than a formal systematic review or meta-analysis. Therefore, the sources were selected based on their clinical and fundamental relevance to the reviewed molecular pathways, resulting in the most pertinent peer-reviewed studies and clinical guidelines being included in the final review.

## 3. Molecular Mechanisms of Physiological Decidualization

Successful embryo implantation requires the coordinated action of progesterone, its receptors, and transcriptional co-factors. The implantation window is a brief period during the mid-secretory phase (days 19–23 of a 28-day cycle) when the endometrium becomes receptive to an embryo [14]. The main event during this time is decidualization, which transforms endometrial stromal cells into specialized secretory decidual cells [15]. This process involves morphological changes, such as cell rounding and the accumulation of glycogen and lipids, along with the activation of specific genes.

Progesterone regulates decidualization by binding to two intracellular receptor isoforms: PRA and PRB. These isoforms have distinct functions: PRB is the primary activator of target gene transcription, while PRA modulates and can inhibit PRB activity [16]. Maintaining the correct ratio between PRA and PRB in endometrial stromal cells is necessary for a normal transcriptional response to progesterone [14].

The transcription factor FOXO1 is a required co-factor that enables the progesterone receptor (PR) to bind to target DNA. In response to progesterone, FOXO1 translocates to the nucleus to activate decidualization genes [17]. Key targets of the FOXO1-PR complex include the IRF4 gene, alongside classic decidualization markers such as insulin-like growth factor-binding protein-1 (IGFBP1) and decidual prolactin (dPRL) [18]. The transcription factor HOXA10 also plays a regulatory role by promoting embryo adhesion and endometrial remodeling [19]. HOXA10 co-regulates the expression of IGFBP1 and dPRL, meaning its suppression directly impairs decidualization [20].

Therefore, normal decidualization depends on adequate progesterone levels, functionally active PR isoforms in the correct ratio, nuclear FOXO1 for PR co-activation, and sufficient HOXA10 expression [14,15,17,19]. Disruption in any of these components reduces endometrial receptivity and drives progesterone resistance.

## 4. Mechanisms of Progesterone Resistance in PCOS/PMOS

### 4.1. Endocrine Factors: Hyperinsulinemia and Hyperandrogenism

The inactivation of transcription factor FOXO1 is a major proposed molecular mechanism of progesterone resistance. In a healthy endometrium, FOXO1 drives stromal cell decidualization and upregulates key markers such as dPRL and IGFBP1 [21]. However, chronic hyperinsulinemia activates the PI3K/AKT pathway, which phosphorylates FOXO1 and confines it to the cytoplasm. Because the progesterone receptor relies on FOXO1 to bind to the IRF4 gene, this cytoplasmic retention essentially disables progesterone signaling [22]. As a result, functional decidualization fails, even if the cells show partial morphological changes [23,24]. In vitro evidence suggests that insulin also impairs the decidual response via the PI3K/AKT-NR4A1 pathway [24].

To better understand how high insulin levels affect the endometrium, Ujvari et al. studied isolated human endometrial stromal cells in vitro. They found that high insulin concentrations activate the PI3K/AKT pathway, which forces FOXO1 out of the cell nucleus and inactivates it. As a result, the expression of key genes required for decidualization, such as IGFBP1, significantly drops [24]. These in vitro findings are supported by tissue analysis from patients with PCOS and hyperinsulinemia. In these patients, FOXO1 remains in its inactive phosphorylated form in the cytoplasm, which is also linked to lower levels of the GLUT4 glucose transporter (SLC2A4) [25]. Ultimately, these in vitro findings suggest that hyperinsulinemia contributes to the disruption of the normal intracellular decidualization process. These findings suggest a mechanistic basis for the limited efficacy of exogenous progesterone in overcoming endometrial resistance, though this interpretation requires further clinical validation.

High androgen levels further contribute to resistance. In PCOS, the endometrium overexpresses androgen receptors (ARs) [26]. Although primarily supported by indirect evidence from other steroid-responsive tissues rather than definitive direct proof in the human endometrium, it is hypothesized that these overexpressed receptors compete with progesterone receptors for shared intracellular co-activators, blunting the overall progesterone response [27].

### 4.2. Immuno-Inflammatory Alterations

Systemic inflammation directly contributes to progesterone resistance. Women with PCOS/PMOS show elevated levels of tumor necrosis factor-alpha (TNF-α), an inflammatory marker of metabolic dysfunction [28,29]. Within the endometrium, the activation of TLR4/NF-κB signaling sustains this chronic inflammation [30,31], forcing decidual cells into secondary senescence and making them unresponsive to progesterone [32]. Observed correlations suggest that excess androgens also contribute to the disruption of the local immune environment by promoting a persistent activation of pro-inflammatory M1 macrophages without a matching increase in anti-inflammatory M2 macrophages [33]. This macrophage imbalance breaks down immune tolerance and prevents successful embryo implantation [33]. Furthermore, high androgen levels impair the AMPK/SIRT1/PDK4 axis, creating an energy deficit in decidualizing cells [34]. Although SIRT1 is essential for normal decidualization [35,36], its precise interaction with FOXO1 remains unclear [37]. AMPK, however, is definitively required for the extracellular matrix remodeling that supports implantation [38].

In addition to classical inflammatory pathways, the process of ferroptosis (an iron-dependent form of programmed cell death) is a contributing factor to endometrial dysfunction in PCOS. Zhang et al. reported that altered levels of dipeptidyl peptidase 4 (DPP4) in the endometrium mediate local tissue damage and disrupt stromal cell decidualization. Therefore, inhibiting the DPP4 pathway limits ferroptosis and improves decidual capacity. This mechanism provides an explanation for progesterone resistance [39].

### 4.3. Epigenetic Reprogramming

MicroRNA dysregulation is a contributing factor to epigenetic reprogramming of the endometrium in women with PCOS. Systemic metabolic aberrations (most notably hyperandrogenism and hyperinsulinemia) alter the expression of microRNA and disrupt post-transcriptional control of genes essential for stromal differentiation [38]. Specifically, these microRNAs target and impair the PI3K/AKT/FOXO1 signaling pathways that are crucial for endometrial remodeling, glucose transport (via GLUT4 modulation), and decidualization. Furthermore, this metabolic dysregulation interferes with normal transcription binding and histone modification, suppressing key markers of endometrial receptivity and leading to progesterone resistance [38,39,40,41].

Epigenetic modifications, including DNA methylation and microRNA dysregulation, stabilize progesterone resistance and enable its heritability. Systemic hypomethylation in PCOS/PMOS affects the ovaries, adipose tissue, and blood, while the methylation profiles of key genes like LHCGR and INSR correlate with the severity of metabolic dysfunction [42]. These modifications disrupt genes involved in steroidogenesis and hormonal regulation [43]. This epigenetic variability explains individual differences in clinical insulin sensitivity and hormonal responses [44]. Because systemic hypomethylation directly correlates with tissue dysfunction [45], specific DNA methylation and microRNA profiles can serve as candidate biomarkers [46].

Recent single-nucleus RNA sequencing (snRNA-seq) provides a detailed cellular atlas of the PCOS endometrium. In stromal cells, these data reveal suppressed estrogen receptor-α (ESR1) expression and disrupted extracellular matrix organization. Evidence from animal models indicates that these epigenetic signatures can pass to the next generation. Metabolic interventions, such as dietary modifications, can restore a normal methylome and prevent the transgenerational transmission of PCOS traits [47].

Single-nucleus RNA sequencing (snRNA-seq) has recently detailed the endometrial transcriptomic profile in PCOS. An analysis of over 240,000 endometrial cells revealed that essential receptivity genes, including HOXA10 and LIF, are downregulated in distinct stromal and epithelial cell populations. This altered expression pattern is closely associated with impaired endometrial receptivity and implantation failure. Furthermore, a 16-week intervention with metformin was shown to partially restore these transcriptomic disruptions, indicating that the molecular mechanisms of progesterone resistance can be modulated metabolically [43].

The proposed pathways of progesterone resistance in PMOS are summarized in Figure 1.

## 5. Candidate Biomarkers of Endometrial Receptivity

In PCOS/PMOS, suppressed decidualization markers, specifically IGFBP1 and dPRL, reflect impaired endometrial receptivity [48,49]. Importantly, however, no established criteria currently exist for diagnosing progesterone resistance, and in clinical practice the condition is inferred mainly from recurrent implantation failure despite adequate serum progesterone levels. This functional deficit results from a severely depleted pool of viable endometrial stromal fibroblasts [45]. The depletion is driven by premature cellular senescence caused by lysophosphatidylcholine acyltransferase 3 (LPCAT3) deficiency and disrupted phosphatidylcholine metabolism. Senescent fibroblasts lack both proliferative and differentiation capacities, which arrests decidualization and prevents implantation. Restoring phospholipid homeostasis reverses this senescence, identifying LPCAT3 as a specific therapeutic target [44].

At the molecular level, normal IGFBP1 expression requires the HBP1 protein, which upregulates progesterone receptor (PR) activity via H3K4me3 modification [47]. The senescence-induced loss of functionally active stromal cells dictates implantation failure in PCOS/PMOS, independent of successful ovulation induction.

Clinically, serum IGFBP1 remains unvalidated as a standalone predictor for IVF outcomes. Non-invasive epigenetic profiling of peripheral leukocytes offers a diagnostic alternative to endometrial biopsies, though routine application requires further clinical validation [47,50].

## 6. Therapeutic Strategies: From Metabolic Correction to Receptivity Restoration

Effective therapy requires restoring target tissue sensitivity rather than simply supplementing hormones. As illustrated in Figure 1, endometrial resistance stems from disrupted intracellular signaling—specifically at the receptor, co-activator, and epigenetic levels—not an absolute hormone deficiency. Interventions must focus on correcting hyperinsulinemia, hyperandrogenism, and chronic inflammation, as these factors sequester FOXO1, disrupt PR isoform ratios, and establish epigenetic barriers.

Therapeutic strategies can be divided into three categories based on the level of evidence: (a) established treatments for the metabolic and reproductive manifestations of PCOS (such as lifestyle modifications, metformin, and inositols), which improve systemic profiles while their direct role in reversing endometrial resistance remains a mechanistic model; (b) interventions with preliminary evidence regarding endometrial receptivity (such as GLP-1 receptor agonists); and (c) experimental epigenetic and metabolic approaches (such as HDAC and SGLT2 inhibitors). The current and emerging therapeutic strategies targeting these specific mechanisms are summarized in Table 2.

Clinical evidence confirms the efficacy of insulin-sensitizing agents in improving reproductive outcomes. A comprehensive meta-analysis of randomized controlled trials involving overweight and obese patients with PCOS demonstrated that metformin administration (1500–2000 mg/day for 3 to 6 months) significantly increased clinical pregnancy rates (OR = 2.31) and live birth rates (OR = 2.11) compared to placebo or no treatment [51]. Similarly, myo-inositol supplementation, typically administered at 4000 mg/day alongside folic acid, effectively restores spontaneous ovulation. In cohorts undergoing in vitro fertilization, myo-inositol pretreatment resulted in a decreased requirement for recombinant FSH and a significantly higher clinical pregnancy rate [52].

Metformin directly targets this metabolic dysfunction. By lowering insulin levels, it reduces FOXO1 phosphorylation via the PI3K/AKT pathway, enabling FOXO1 nuclear translocation and PR-dependent transcription [53]. Metformin also modulates microRNA expression to improve endometrial receptivity [54]. While some recent meta-analyses indicate that continuing metformin in the first trimester may reduce miscarriage risk and improve birth rates [54], current international evidence-based guidelines do not universally recommend routine continuation of metformin during pregnancy, emphasizing that treatment decisions should be individualized [51,53].

Although inositols (such as myo-inositol or specific myo-inositol-to-D-chiro-inositol ratios) are widely used as supportive treatment for patients intolerant to metformin [55], the clinical evidence regarding optimal doses and ratios, as well as the reproductive outcomes, shows considerable heterogeneity. Therefore, they should be viewed as supplementary treatment that may improve androgen profiles, insulin resistance, and ovulation and pregnancy rates [56].

GLP-1 receptor agonists, including liraglutide and semaglutide, according to current clinical protocols, must be used strictly as preconceptional treatment for weight management and insulin level improvement, requiring reliable contraception and complete discontinuation 4–8 weeks prior to attempting conception or embryo transfer [57,58]. Any potential beneficial effects on endometrial tissue remain entirely hypothetical and secondary to systemic metabolic changes; therefore, these drugs require complete discontinuation 4–8 weeks prior to conception [59,60]. Lifestyle modification is the first-line intervention; a 5–10% weight reduction effectively upregulates endometrial receptivity markers [61,62].

### Hypothesis-Generating Experimental Targets

Beyond established therapies, experimental approaches target specific epigenetic and signaling pathways, such as histone deacetylase (HDAC) and SGLT2 inhibitors [60,61,62]. However, current evidence comes mostly from in vitro endometrial cancer models; these findings cannot be directly applied to PCOS/PMOS [63,64]. Given this significant gap, their actual clinical effectiveness in overcoming endometrial resistance requires dedicated prospective validation.

## 7. Future Directions

Addressing progesterone resistance in PMOS requires translating recent molecular insights into clinical applications through targeted investigations:

First, validation of non-invasive diagnostic tools is necessary. Prospective, large-scale cohort studies should evaluate whether leukocyte-derived microRNA profiles can reliably serve as blood-based candidate biomarkers for endometrial receptivity. Validated signatures could reduce the reliance on endometrial biopsies, allowing for non-invasive monitoring of the implantation window and personalized transfer timing.

Second, clinical evaluation of novel therapeutics must advance. Well-designed Phase II and III clinical trials are needed to assess emerging metabolic and epigenetic agents, such as SGLT2 inhibitors and histone deacetylase (HDAC) inhibitors, in well-defined cohorts of patients with recurrent implantation failure. These studies should prioritize standard reproductive endpoints—clinical pregnancy and live birth rates—alongside molecular markers of successful decidualization, including IGFBP1 and PRL expression.

Finally, pharmaceutical development should focus on tissue-specific delivery methods. Restoring the physiological balance between progesterone receptor isoforms A and B directly within the endometrium remains a key challenge. Developing targeted local delivery systems could correct this receptor imbalance without causing systemic endocrine side effects, offering a direct therapeutic approach for progesterone resistance in PMOS.

## 8. Discussion

Endometrial dysfunction and implantation failure in PCOS/PMOS are multifactorial and driven by endocrine, immuno-inflammatory, and epigenetic dysregulation. Within this complex mechanism, progesterone resistance serves as a contributing factor rather than a sole cause, explaining the limited efficacy of exogenous progesterone therapy.

In routine practice, progesterone resistance in PCOS/PMOS should be managed through metabolic correction—lifestyle modification, metformin, and inositols—combined with individualized IVF protocols [40]. Where GLP-1 receptor agonists are used, they should be confined to the preconceptional period and stopped several weeks before any attempt at conception or embryo transfer [57]. Direct modulators of resistance pathways, including HDAC, PI3K, and SGLT2 inhibitors, remain experimental and should be offered only within clinical trials. Until targeted therapies are validated, optimized metabolic control remains the only evidence-based strategy for improving endometrial receptivity in these patients.

Current guidelines do not yet recognize progesterone resistance as a distinct clinical target, leaving a practical gap between molecular findings and routine clinical care. Future research must align with biomarker-driven patient stratification [41]. Data on epigenetic heritability come entirely from animal models and cannot be directly applied to human transgenerational transmission. Similarly, while various molecular signatures are being explored as potential markers, there is still not enough clinical validation to recommend epigenetic stratification of patients with PCOS in routine practice. Clinical trials are also necessary to determine if targeted treatments, such as HDAC/PI3K or SGLT2 inhibitors, improve endometrial receptivity. Recognizing progesterone resistance in clinical practice is a necessary step to move from generalized IVF protocols to personalized, phenotype-specific therapies.

Ultimately, the transition of PCOS to PMOS highlights the systemic metabolic nature of this condition, suggesting that standard IVF protocols may no longer be sufficient. Clinical decision-making and treatment choices may require a shift toward personalized strategies based on the patient’s specific metabolic phenotype, inflammatory status, and previous responses to therapy (Figure 2).

Declaration of Generative AI and AI-Assisted Technologies: During the preparation of this work, the authors used generative AI technology strictly for language editing and formatting assistance to improve the English readability of the manuscript. After using this tool, the authors rigorously reviewed and edited the content, taking full responsibility for the scientific accuracy and integrity of the publication.

## Figures and Tables

**Figure 1:**
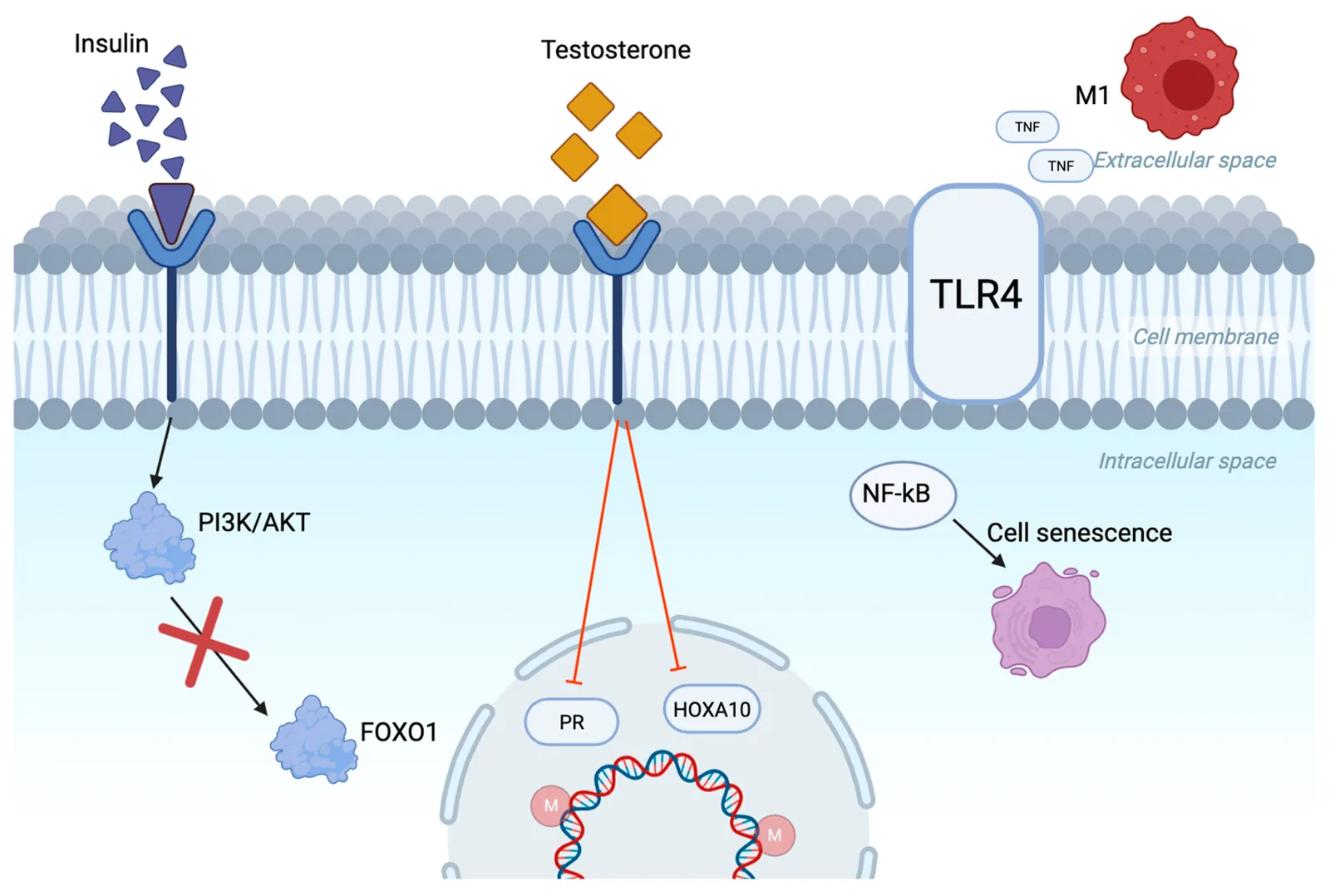
Molecular mechanisms of endometrial progesterone resistance in PCOS/PMOS. Pathogenesis involves three core axes: (1) endocrine (hyperinsulinemia sequesters FOXO1; hyperandrogenism suppresses PR and HOXA10), (2) immuno-inflammatory (M1 macrophage shift, NF-κB activation, and cellular senescence), and (3) epigenetic (DNA hypomethylation stabilizing the resistant phenotype).

**Figure 2:**
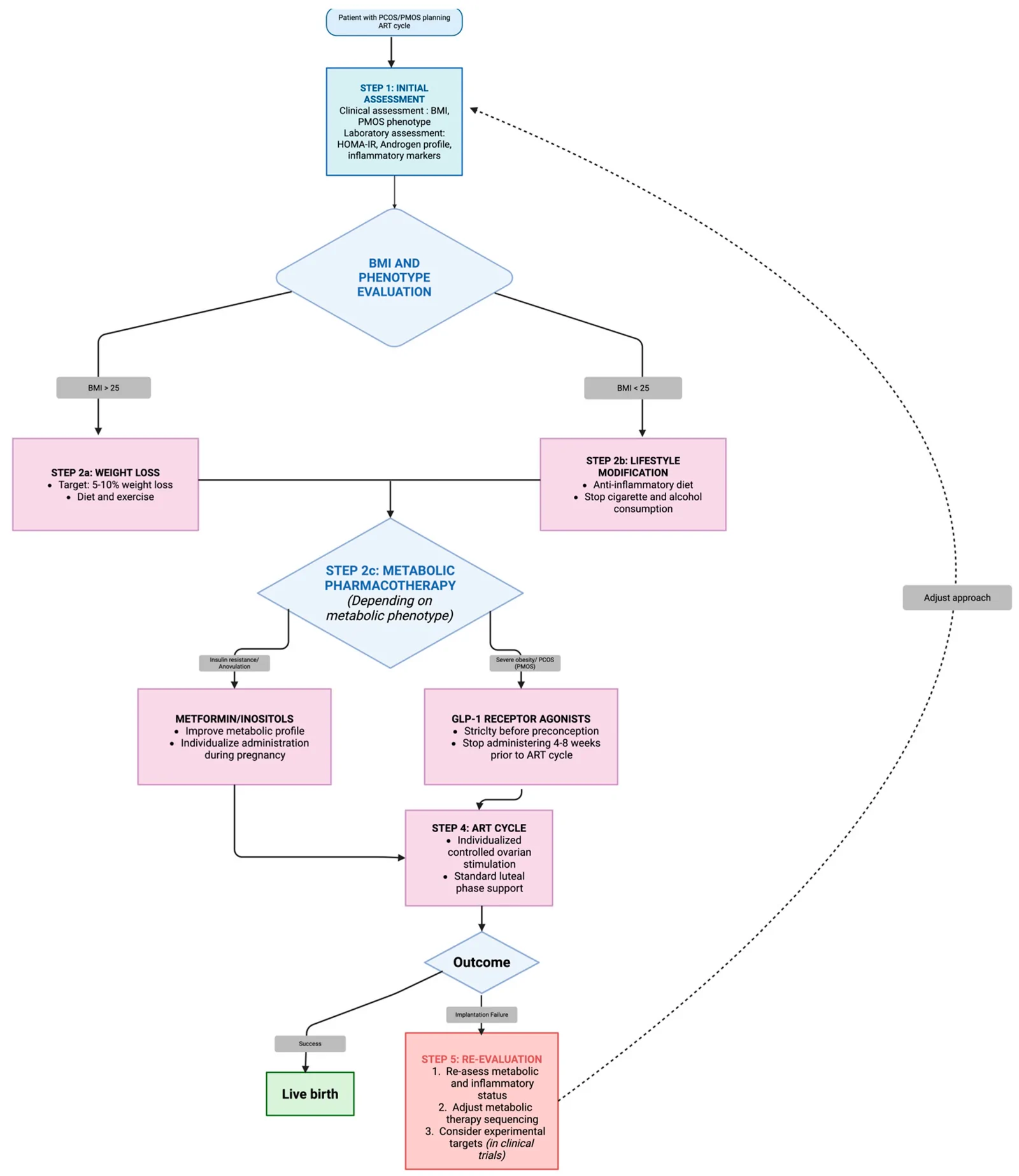
Management of PCOS/PMOS patients undergoing ART. The algorithm outlines concurrent lifestyle and metabolic therapies based on individual phenotypes, followed by individualized ART protocols and reassessment strategies for implantation failure.

**Table 1:** Historical evolution of the conceptualization and primary therapeutic approaches for PCOS/PMOS.

Era/Milestone	Primary Conceptualization	Therapeutic Approach
1935 (Stein & Leventhal)	Gynecological disorder (anovulation and bilateral polycystic ovaries)	Surgical interventions (e.g., ovarian wedge resection)
Mid-to-Late 20th Century	Neuroendocrine dysfunction (altered gonadotropins and steroidogenesis)	Hormonal ovulation induction (e.g., clomiphene citrate)
1980s–1990s	Systemic metabolic disorder (hyperinsulinemia and insulin resistance)	Metabolic correction using insulin-sensitizing agents (e.g., metformin, inositols)
2026	Polycystic Ovary Morphology and Metabolic Syndrome (PMOS)	Personalized metabolic targeted therapies to restore endometrial sensitivity and overcome progesterone resistance

**Table 2:** Established and experimental therapeutic strategies for overcoming progesterone resistance in PCOS/PMOS.

Therapeutic Strategy	Evidence Level	Primary Molecular Target/Mechanism	Clinical Impact & Status
Metformin	A	Reduces FOXO1 phosphorylation via PI3K/AKT pathway; modulates microRNA	First-line metabolic correction
Inositols (Myo/D-chiro)	A	Improves androgen profiles and insulin resistance (HOMA-IR)	Supplementary treatment that may improve androgen profiles, insulin resistance, and ovulation and pregnancy rates
Lifestyle Modification	A	Broad metabolic reset (via 5–10% weight reduction)	Achieves preconceptional weight loss
GLP-1 Agonists	B	Systemic weight loss and improved insulin dynamics	Requires discontinuation 4–8 weeks prior to conception
HDAC Inhibitors	C	Epigenetic modulation; counters PI3K/AKT hyperactivation	Preclinical investigation; requires direct clinical validation for overcoming endometrial resistance

А—established clinical evidence; B—preliminary clinical evidence; С—preclinical/experimental evidence.

## Data Availability

The original contributions presented in this study are included in the article. Further inquiries can be directed to the corresponding authors.

## Abbreviations

The following abbreviations are used in this manuscript:

AR	Androgen receptor
dPRL	Decidual prolactin
FOXO1	Forkhead box protein O1
GLP-1	Glucagon-like peptide-1
HDAC	Histone deacetylase
IGFBP1	Insulin-like growth factor-binding protein 1
PCOS	Polycystic ovary syndrome
PMOS	Polycystic ovary morphology and metabolic syndrome
PR	Progesterone receptor (PRA and PRB isoforms)
SGLT2	Sodium–glucose cotransporter 2
TNF-α	Tumor necrosis factor-alpha

## References

[1] 1 in 6 People Globally Affected by Infertility: WHO. https://www.who.int/news/item/04-04-2023-1-in-6-people-globally-affected-by-infertility.

[2] Yasmin A., Roychoudhury S., Paul Choudhury A., Ahmed A.B.F., Dutta S., Mottola F., Verma V., Kalita J.C., Kumar D., Sengupta P. (2022). Polycystic Ovary Syndrome: An Updated Overview Foregrounding Impacts of Ethnicities and Geographic Variations. Life.

[3] Insulin Resistance in Polycystic Ovary Syndrome: Pathophysiological Mechanisms of Menstrual Dysfunction and Evidence-based Treatment Strategies. https://academic.oup.com/biolreprod/article-abstract/113/6/1340/8244406.

[4] Matsuyama S., Whiteside S., Li S.-Y. (2024). Implantation and Decidualization in PCOS: Unraveling the Complexities of Pregnancy. Int. J. Mol. Sci..

[5] Stein I.F., Leventhal M.L. (1935). Amenorrhea Associated with Bilateral Polycystic Ovaries. Am. J. Obstet. Gynecol..

[6] Wedge Resection for Polycystic Ovaries: A Critical Analysis of 40 Operations. https://www.nejm.org/doi/full/10.1056/NEJM195408192510802.

[7] Clomiphene Citrate for Ovulation Induction (Chapter 23)—Infertility and Assisted Reproduction. https://www.cambridge.org/core/books/abs/infertility-and-assisted-reproduction/clomiphene-citrate-for-ovulation-induction/BBC4B26CB803E6A976BA9DF8893F1F2A.

[8] Witchel S.F., Azziz R., Oberfield S.E. (2022). History of Polycystic Ovary Syndrome, Premature Adrenarche, and Hyperandrogenism in Pediatric Endocrinology. Horm. Res. Paediatr..

[9] High Androgen Level During Controlled Ovarian Stimulation Cycle Impairs Endometrial Receptivity in PCOS Patients. https://www.nature.com/articles/s41598-024-74295-7.

[10] Polyendocrine Metabolic Ovarian Syndrome, the New Name for Polycystic Ovary Syndrome: A Multistep Global Consensus Process. https://www.thelancet.com/journals/lancet/article/PIIS0140-6736%2826%2900717-8/fulltext.

[11] Matorras R., Pijoan J.I., Laínz L., Díaz-Nuñez M., Sainz H., Pérez-Fernandez S., Moreira D. (2022). Polycystic ovarian syndrome and miscarriage in IVF: Systematic revision of the literature and meta-analysis. Arch. Gynecol. Obstet..

[12] Progesterone Resistance in PCOS Endometrium: A Microarray Analysis in Clomiphene Citrate-Treated and Artificial Menstrual Cycles. https://pubmed.ncbi.nlm.nih.gov/21411543/.

[13] Li X., Feng Y., Lin J.F., Billig H., Shao R. (2014). Endometrial progesterone resistance and PCOS. J. Biomed. Sci..

[14] Vasquez Y., DeMayo F. (2013). Role of Nuclear Receptors in Blastocyst Implantation. Seminars in Cell & Developmental Biology.

[15] Lessey B.A., Young S.L. (2019). What exactly is endometrial receptivity?. Fertil. Steril..

[16] DeMayo F.J., Lydon J.P. (2020). 90 YEARS OF PROGESTERONE: New insights into progesterone receptor signaling in the endometrium required for embryo implantation. J. Mol. Endocrinol..

[17] Vasquez Y.M., Mazur E.C., Li X., Kommagani R., Jiang L., Chen R., Lanz R.B., Kovanci E., Gibbons W.E., DeMayo F.J. (2015). FOXO1 is required for binding of PR on IRF4, novel transcriptional regulator of endometrial stromal decidualization. Mol. Endocrinol..

[18] Du H., Taylor H.S. (2015). The Role of Hox Genes in Female Reproductive Tract Development, Adult Function, and Fertility. Cold Spring Harb. Perspect. Med..

[19] FoxO1 Is a Cell-Specific Core Transcription Factor for Endometrial Remodeling and Homeostasis During Menstrual Cycle and Early Pregnancy. https://academic.oup.com/humupd/article/27/3/570/6090095.

[20] Ujvari D., Jakson I., Babayeva S., Salamon D., Rethi B., Gidlöf S., Hirschberg A.L. (2017). Dysregulation of In Vitro Decidualization of Human Endometrial Stromal Cells by Insulin via Transcriptional Inhibition of Forkhead Box Protein O1. PLoS ONE.

[21] Kohan K., Carvajal R., Gabler F., Vantman D., Romero C., Vega M. (2010). Role of the transcriptional factors FOXO1 and PPARG on gene expression of SLC2A4 in endometrial tissue from women with polycystic ovary syndrome. Reproduction.

[22] Apparao K.B.C., Lovely L.P., Gui Y., Lininger R.A., Lessey B.A. (2002). Elevated endometrial androgen receptor expression in women with polycystic ovarian syndrome. Biol. Reprod..

[23] Hosseinzadeh P., Barsky M., Gibbons W.E., Blesson C.S. (2021). Polycystic Ovary Syndrome and the Forgotten Uterus. F S Rev..

[24] Cermik D., Selam B., Taylor H.S. (2003). Regulation of HOXA-10 expression by testosterone in vitro and in the endometrium of patients with polycystic ovary syndrome. J. Clin. Endocrinol. Metab..

[25] Xie Q., Xiong X., Xiao N., He K., Chen M., Peng J., Su X., Mei H., Dai Y., Wei D. (2019). Mesenchymal Stem Cells Alleviate DHEA-Induced Polycystic Ovary Syndrome (PCOS) by Inhibiting Inflammation in Mice. Stem Cells Int..

[26] Verma B., Verma P., Nair S.S., Bosco S.J., Kathiresan S., Kshatri A.H.S., Tiwari A.K., Kenche A.R., Bashichandra P., Kumar S.K.I. (2025). Tumor Necrosis Factor-Alpha (TNF-α) Levels in Women With Polycystic Ovary Syndrome (PCOS): A Systematic Review and Meta-Analysis of Observational Studies. Cureus.

[27] Raheem B.M., Obaid R.M., Ali B.R., Al-Fahham A.A. (2024). The role of TNF-α and il-6 SNP in polycystic ovary syndrome susceptibility. Pol. Merkur. Lek..

[28] Hong L., Xiao S., Diao L., Lian R., Chen C., Zeng Y., Liu S. (2024). Decreased AMPK/SIRT1/PDK4 induced by androgen excess inhibits human endometrial stromal cell decidualization in PCOS. Cell Mol. Life Sci..

[29] Hwang Y.J., Sung G.-J., Marquardt R., Young S.L., Lessey B.A., Kim T.H., Cheon Y.P., Jeong J.W. (2022). SIRT1 plays an important role in implantation and decidualization during mouse early pregnancy. Biol. Reprod..

[30] Cummings M.J., Yu H., Paudel S., Hu G., Li X., Hemberger M., Wang X. (2022). Uterine-specific SIRT1 deficiency confers premature uterine aging and impairs invasion and spacing of blastocyst, and stromal cell decidualization, in mice. Mol. Hum. Reprod..

[31] Yu S.-L., Lee S.-I., Park H.-W., Lee S.K., Kim T.-H., Kang J., Park S.R. (2022). SIRT1 suppresses in vitro decidualization of human endometrial stromal cells through the downregulation of forkhead box O1 expression. Reprod. Biol..

[32] Zhong Y., Ding J., Xia F. (2025). DNA methylation and its impact on ovarian steroidogenesis in women with polycystic ovary syndrome: Insights from human and animal models. Arch. Gynecol. Obstet..

[33] Hu R., Jin L., Li Q., Fan P., Fan W., Xu J. (2026). Epigenetic Regulation by DNA Methylation in PCOS and POI: Mechanisms of Ovarian Dysfunction and Implications for Clinical Research. J. Ovarian Res..

[34] Kobayashi H., Shigetomi H., Nishio M., Umetani M., Imanaka S., Hashimoto H. (2026). Molecular Mechanisms Underlying Reproductive Dysfunction in Polycystic Ovary Syndrome. Reprod. Sci..

[35] Bhingardeve S., Sagvekar P., Mukherjee S. (2025). Altered DNA methylation and miRNA expression: Epi-marks for elucidating the pathophysiology of PCOS. Reprod. Biomed. Online.

[36] Zhang J., Wang R., Tian X., Ding N., He Y., Wang K., Tao C., Cai J., Jian Q., Wang F. (2025). The mechanism study of targeting DPP4 in regulating ferroptosis and its influence on endometrial receptivity in PCOS. Biol. Sex Differ..

[37] Zhang J., Xing C., Cheng X., He B. (2022). Canagliflozin combined with metformin versus metformin monotherapy for endocrine and metabolic profiles in overweight and obese women with polycystic ovary syndrome: A single-center, open-labeled prospective randomized controlled trial. Front. Endocrinol..

[38] Xu R., Wang Z. (2021). Involvement of Transcription Factor FoxO1 in the Pathogenesis of Polycystic Ovary Syndrome. Front. Physiol..

[39] Huang Y., Zhu Q., Sun Y. (2025). Glucose metabolism and endometrium decidualization. Front. Endocrinol..

[40] Teede H.J., Tay C.T., Laven J.J.E., Dokras A., Moran L.J., Piltonen T.T., Costello M.F., Boivin J., Redman L.M., Boyle J.A. (2023). Recommendations From the 2023 International Evidence-based Guideline for the Assessment and Management of Polycystic Ovary Syndrome. J. Clin. Endocrinol. Metab..

[41] Teede H.J., Gibson M., Laven J., Dokras A., Moran L.J., Piltonin T., Costello M., Mousa A., Joham A.E., Tay C.T. (2024). International PCOS guideline clinical research priorities roadmap: A co-designed approach aligned with end-user priorities in a neglected women’s health condition. eClinicalMedicine.

[42] Cao P., Li H., Wang P., Zhang X., Guo Y., Zhao K., Guo J., Li X., Nashun B. (2024). DNA Hypomethylation-Mediated Transcription Dysregulation Participates in Pathogenesis of Polycystic Ovary Syndrome. Am. J. Pathol..

[43] Anania C. (2025). The PCOS-endometrial cell atlas. Nat. Genet..

[44] Ray R.P., Padhi M., Jena S., Patnaik R., Rattan R., Nayak A.K. (2022). Study of Association of Global Deoxyribonucleic Acid Methylation in Women with Polycystic Ovary Syndrome. J. Hum. Reprod. Sci..

[45] Li C., Cheng D., Ren H., Zhang T. (2025). Unraveling the gut microbiota’s role in PCOS: A new frontier in metabolic health. Front. Endocrinol..

[46] Cerván-Martín M., Suazo-Sánchez M.I., Rivera-Egea R., Garrido N., Luján S., Romeu G., Santos-Ribeiro S., Castilla J.A., Gonzalvo M.C., Clavero A. (2020). Intronic variation of the SOHLH2 gene confers risk to male reproductive impairment. Fertil. Steril..

[47] Kawa I.A., Fatima Q., Masood A., Mir S.A., Mir I., Manzoor S., Rashid F. (2026). Diagnostic and prognostic evaluation of miRNA-93 and miRNA-223 in women with polycystic ovary syndrome. Gene Rep..

[48] Tamura I., Asada H., Maekawa R., Tanabe M., Lee L., Taketani T., Yamagata Y., Tamura H., Sugino N. (2012). Induction of IGFBP-1 expression by cAMP is associated with histone acetylation status of the promoter region in human endometrial stromal cells. Endocrinology.

[49] Regulation of Decidualization, Interleukin-11 and Interleukin-15 by Homeobox A 10 in Endometrial Stromal Cells. https://www.researchgate.net/publication/44607657_Regulation_of_decidualization_interleukin-11_and_interleukin-15_by_homeobox_A_10_in_endometrial_stromal_cells.

[50] Yi P., Qi Y., Mao S., Cao Y., Zhou Y., Fu X. (2026). An endometrial tissue-based predictive model for polycystic ovary syndrome constructed from immuno-metabolic dysregulation features mediated by ACO1. J. Ovarian Res..

[51] Guan Y., Wang D., Bu H., Zhao T., Wang H. (2020). The Effect of Metformin on Polycystic Ovary Syndrome in Overweight Women: A Systematic Review and Meta-Analysis of Randomized Controlled Trials. Int. J. Endocrinol..

[52] Facchinetti F., Unfer V., Dewailly D., Kamenov Z.A., Diamanti-Kandarakis E., Laganà A.S., Nestler J.E., Soulage C.O. (2020). Inositols in Polycystic Ovary Syndrome: An Overview on the Advances. Trends Endocrinol. Metab..

[53] Zhai J., Yao G.-D., Wang J.-Y., Yang Q.-L., Wu L., Chang Z.-Y., Sun Y.-P. (2019). Metformin Regulates Key MicroRNAs to Improve Endometrial Receptivity Through Increasing Implantation Marker Gene Expression in Patients with PCOS Undergoing IVF/ICSI. Reprod. Sci..

[54] Cheshire J., Garg A., Smith P., Devall A.J., Coomarasamy A., Dhillon-Smith R.K. (2025). Preconception and first-trimester metformin on pregnancy outcomes in women with polycystic ovary syndrome: A systematic review and meta-analysis. Am. J. Obstet. Gynecol..

[55] Duan M., Yang M., Li C., Wu X., Yin X., Zhu H.H. (2026). Effects of inositol in women with polycystic ovary syndrome: An umbrella review of meta-analyses from randomized controlled trials. Front. Endocrinol..

[56] Fitz V., Graca S., Mahalingaiah S., Liu J., Lai L., Butt A., Armour M., Rao V., Naidoo D., Maunder A. (2024). Inositol for Polycystic Ovary Syndrome: A Systematic Review and Meta-analysis to Inform the 2023 Update of the International Evidence-based PCOS Guidelines. J. Clin. Endocrinol. Metab..

[57] Becker A.S., de Castilhos J.P., Shugair S.A.S., Colombo T., Hentschke M.-R. (2026). GLP-1 Receptor Agonists and Fertility: What Is Known So Far?. JBRA Assist. Reprod..

[58] Turlej K., Kowalczuk D., Kasianik V., Lazitskaya D., Sobchynskyi M., Milasheuskaya V., Myrnyi A., Kiełbaszewska I., Surosz N., Miraniuk K. (2025). The Potential Role of Glucagon-Like Peptide-1 Receptor Agonists in Polycystic Ovary Syndrome: A Narrative Review. Qual. Sport.

[59] The Safety and Efficacy of Liraglutide Combined with Metformin in Clinical Treatment of Polycystic Ovary Syndrome Patients: A Meta-Analysis. https://link.springer.com/article/10.1186/s12905-025-03787-z.

[60] Sola-Leyva A., Pathare A.D.S., Apostolov A., Aleksejeva E., Kask K., Tammiste T., Ruiz-Durán S., Risal S., Acharya G., Salumets A. (2025). The hidden impact of GLP-1 receptor agonists on endometrial receptivity and implantation. Acta Obstet. Gynecol. Scand..

[61] Abdulkhalikova D., Sustarsic A., Vrtačnik Bokal E., Jancar N., Jensterle M., Burnik Papler T. (2022). The Lifestyle Modifications and Endometrial Proteome Changes of Women With Polycystic Ovary Syndrome and Obesity. Front. Endocrinol..

[62] Endometrial Transcriptome Alterations Following Short-Term Liraglutide Treatment in Infertile Women with Polycystic Ovary Syndrome and Obesity. https://www.endocrine-abstracts.org/ea/0099/ea0099rc1.2.

[63] Zhang X., Lazaro-Camp V., Li T., Qi A., Lan L., Lamont L., Zhao L., Meehan M.R., Gardner S.N., Meng W. (2025). Dual PI3K and HDAC inhibitor, CUDC-907, effectively inhibits endometrial cancer growth in vitro and in vivo. Front. Oncol..

[64] Yang Y., Zhou J., Lv Q., Ni Q., Hu B., Wang Y., Qu S., Li G., Yang W., Zhong R. (2025). Canagliflozin alleviates progestin resistance by suppressing RARβ/CRABP2 signaling in THRB knockout endometrial cancer cells. Front. Pharmacol..

